# The influence of power and actor relations on priority setting and resource allocation practices at the hospital level in Kenya: a case study

**DOI:** 10.1186/s12913-016-1796-5

**Published:** 2016-09-30

**Authors:** Edwine W. Barasa, Susan Cleary, Mike English, Sassy Molyneux

**Affiliations:** 1 KEMRI Centre for Geographic Medicine Research – Coast, and Wellcome Trust Research Programme, P.O Box 43640-00200 Nairobi, Kenya; 2 Health Economics Unit, University of Cape Town, Cape Town, South Africa; 3 Centre for Tropical Medicine, University of Oxford, Oxford, UK; 4 Department of Pediatrics, University of Oxford, Oxford, UK

**Keywords:** Priority setting, Power practices, Actor interfaces, Hospitals, Kenya

## Abstract

**Background:**

Priority setting and resource allocation in healthcare organizations often involves the balancing of competing interests and values in the context of hierarchical and politically complex settings with multiple interacting actor relationships. Despite this, few studies have examined the influence of actor and power dynamics on priority setting practices in healthcare organizations. This paper examines the influence of power relations among different actors on the implementation of priority setting and resource allocation processes in public hospitals in Kenya.

**Methods:**

We used a qualitative case study approach to examine priority setting and resource allocation practices in two public hospitals in coastal Kenya. We collected data by a combination of in-depth interviews of national level policy makers, hospital managers, and frontline practitioners in the case study hospitals (*n* = 72), review of documents such as hospital plans and budgets, minutes of meetings and accounting records, and non-participant observations in case study hospitals over a period of 7 months. We applied a combination of two frameworks, Norman Long’s *actor interface analysis* and VeneKlasen and Miller’s *expressions of power* framework to examine and interpret our findings

**Results:**

The interactions of actors in the case study hospitals resulted in socially constructed interfaces between: 1) senior managers and middle level managers 2) non-clinical managers and clinicians, and 3) hospital managers and the community. Power imbalances resulted in the exclusion of middle level managers (in one of the hospitals) and clinicians and the community (in both hospitals) from decision making processes. This resulted in, amongst others, perceptions of unfairness, and reduced motivation in hospital staff. It also puts to question the legitimacy of priority setting processes in these hospitals.

**Conclusions:**

Designing hospital decision making structures to strengthen participation and inclusion of relevant stakeholders could improve priority setting practices. This should however, be accompanied by measures to empower stakeholders to contribute to decision making. Strengthening soft leadership skills of hospital managers could also contribute to managing the power dynamics among actors in hospital priority setting processes.

**Electronic supplementary material:**

The online version of this article (doi:10.1186/s12913-016-1796-5) contains supplementary material, which is available to authorized users.

## Background

Given the scarcity of resources, priority setting and resource allocation (PSRA) has emerged as a key health system challenge [1–3]. PSRA is particularly challenging in developing countries, where the gap between healthcare needs and available resources is wide. The judicious use of resources through appropriate PSRA has therefore been considered a key determinant of health system performance [4].

PSRA in healthcare often involves the balancing of competing interests and values in the context of hierarchical and politically complex settings with multiple interacting actor relationships [1]. In such settings, the capacity of different actors to participate effectively in PSRA processes is often influenced by the power dynamics manifested in their relationships [1]. Power - defined as the ability to influence others - is typically derived from varied sources including positional authority, professional status, knowledge and skills, control over resources and physical abilities [5–7]. Power in its various forms tends to manifest itself in such highly political processes such as PRSA. In his seminal work, Steven Lukes (1974) describes what he calls the three dimensions of power [8, 9]. The one dimensional view sees power as the ability of one actor to get another actor to do something they do not want to do. Power in the first dimension is therefore embodied in “concrete decisions” [8, 9]. The second dimension considers “what does not happen” or is “hidden” from decision-making settings and includes the deliberate and conscious exclusion of issues from the agenda. Power is hence exercised through control of the agenda [8, 9]. In the third dimension of power, Lukes argues that power can also operate at a deeper more invisible level [8, 9]. The third dimension of power consists of political socialization that is deeply ingrained such that actors unwittingly follow the dictates of power even against their best interests [8, 9]. In this paper, we examine power in all its three dimensions. While top down models of implementation view power as the control of actors by those with authority at the higher levels of a bureaucracy, bottom up models focus on the micro- practices of power and actor dynamics between and within organizations [10]. By micro-practices, we mean the manifestations of power from the detailed actions and interactions of actors at the frontline of the system (service delivery interface), rather than higher up the system.

While power has been shown to be influential in health policy formulation and implementation [10–12], the influence of power in PSRA processes has seldom been explored in the literature [12]. This is surprising, given that PSRA processes are inherently political, involving a range of actors arbitrating between competing needs and interests [13]. Further, politics has been proposed as a key frame or lens through which organizations (such as hospitals) can be examined and understood [7]. The political frame views organizations as turbulent arenas where ongoing contests of actor interests play out [7]. From this perspective, every significant organizational process is inherently political [7]. From the political frame, the most important decisions in organizations involve allocating scarce resources [7]. Scarcity of resources and enduring differences put conflict at the center of day-to-day dynamics within organizations and make power the most important asset [7].

In this paper, we present findings of case study research to examine the influence of power and actor dynamics on priority setting practices in first referral public hospitals, known as county hospitals, in Kenya. Kenya has a devolved system of governance, with a central government and 47 semi-autonomous units called counties [14]. Under this new governance structure, the health system is structured such that the central Ministry of Health (MOH) has policy making and regulatory roles while responsibilities such as allocation and managing health care resources and service provision are held by county health systems [14]. The healthcare delivery system is organized into four tiers, namely community, primary care, county referral and national referral. Community health services include all community based demand creation activities that are guided by the Kenyan Ministry of Health (MOH) community strategy [15, 16]. Primary healthcare include services provided by public and private maternity homes, health centers and dispensaries. County referral services include first level referral hospitals that are managed by a given county. These are referred to as county hospitals and are the focus of this study. National referral services are comprised of national level facilities, where tertiary referral services are provided.

This paper makes a contribution to the literature on the influence of power in policy implementation by exploring how micro-practices of power shape PSRA practices at the meso level of the health system, county hospitals in Kenya. This paper adopts a bottom-up approach in exploring the practices of power in implementation using a case study design to explore the sources, nature and manifestations of power and its influence on the implementation of PSRA processes in these settings.

## Methods

This paper is based on the analysis of data collected as part of a wider study conducted to describe and evaluate PSRA practices in first referral public hospitals (county hospitals) in Kenya. The case study approach was employed given its appropriateness in examining complex social phenomena [17, 18]. Two county hospitals were purposely selected as cases for the study. The number (2) was informed by the need to conduct an in-depth study within the available resources and timeframe. Two hospital cases were selected purposefully guided by the following criteria: 1) 1^st^ level referral hospitals that were designated as county hospitals; 2) hospitals with a high local resource level and those with a low local resource level; 3) hospitals which had prior relationships our research institution. This last criterion was important because the subject of priority setting was likely to be viewed as political and sensitive. By identifying hospitals with prior contact/relationship or linkage with our institution, we aimed to minimize trust concerns. The selection of hospital cases aimed to identify hospitals that were rich in information – with varying characteristics and experiences - as opposed to representativeness. To maintain confidentiality and minimize the potential of identification of study participants, the hospitals selected for the study will be identified as Hospital A and B.

Within each case study hospital, we selected three PSRA activities as nested cases for in-depth study. We used three criteria to select priority-setting activities: 1) availability and reliability of information; 2) a clearly defined beginning and end to the activity; and 3) full consent to examine the priority-setting case from the hospital. Based on these criteria, we selected the following three PSRA activities: 1) the Hospital Budgeting and annual work planning process; 2) medicine selection decisions in the hospital; and 3) nursing allocation to hospital departments in the hospital.

Data were collected by the first author (EB) who is an experienced health systems researcher with training in qualitative data collection and analysis methods. EB had no prior relationship with all but 1 (former schoolmate) the researcher participants. The participants were verbally informed and provided with a written information sheet that outlined the nature and objectives of the study. Data were collected through a combination of in-depth interviews with hospital managers and frontline workers; non-participant observations over a period of 7 months; and a review of relevant documents including hospital plans, budgets and minutes of meetings. We used in-depth interviews to collect information about the identified priority setting practices from the perspective and experiences of the hospital decision makers, front line practitioners and national level informants. In depth interviews employed topic guides that asked questions about the identity, roles, influence and interests of actors in priority setting processes (Additional file 1). The interviews lasted generally between 30 and 40 min and were carried out in the case study hospitals. Interviews were audio recorded and supplemented by note taking. We reviewed documents relevant to the priority setting activities selected for the study, including the hospital 5 year investment plan, hospital annual work plans (AWPs), hospital quarterly budgets, and minutes from hospital management committee (HMC), hospital management team (HMT), executive expenditure committee (EEC) and medicines and therapeutic committee (MTC) meetings. We also reviewed accounting records and documents recommended by key informants as likely to contain relevant information. These documents were selected for two financial years (2011 – 2012 and 2012 – 2013). We used document review data abstraction guides (Additional file 1) to extract relevant data from the selected documents. EB spent 3.5 months in each of the case study hospitals conducting non-participant observations. These took the form of sitting in and observing hospital quarterly budgeting meetings, AWP meetings, senior management meetings and medicines and therapeutic committee meetings. EB also spent time with and interacted with hospital workers, and held informal discussions with them about priority setting practices in the hospital. During this time, EB spent time in key departments/offices where priority setting activities took place such as the hospital administrator’s office, the accounts department, the pharmacy department and the nursing department. An overt approach to observation was adopted: that is, the hospital staff members were aware of EB’s presence and the objectives of the study. However, beyond brief introductions at the start of the meetings, an unobtrusive approach to observation was employed. A free note-taking approach aided by an observation checklist (Additional file 1) was used.

The selection of participants for interviews was purposive with the aim of selecting individuals who had in-depth knowledge of the identified PSRA activities, and those who took part in or were affected by these PSRA activities. This included senior and middle level hospital managers, frontline practitioners and key informants within the planning departments of the central ministry of health. Participants were selected and interviewed until saturation was reached. In total, 72 participants were selected, 35 from Hospital A, 32 from Hospital B and 5 from the central Ministry of Health. Table 1 outlines the number of participants selected for interviews in each hospital under each category.

**Table 1:** Number of participants selected in each hospital under each category

National-level key informants	5 (all male)	
	Hospital A	Hospital B
Senior managers	6 (2 female, 4 male)	6 (2 female, 4 male)
Mid-level managers	22 (13 male, 9 female)	19 (11 male, 7 female)
Front-line practitioners	7 (3 male, 4 female)	8 (4 male, 4 female)
Hospital sub-total	35 (18 male, 17 female)	32 (17 male, 15 female)
Study total	72 (40 male, 32 female)	

### Application of theory

To explore power relations in the case study hospitals, we integrated and employed two complementary frameworks of power and actor relations. The first of these is Long’s [19] ‘actor interface analysis’ which argues that power dynamics often “fracture” social systems along interfaces that differentiate one group from another based on their power differences. These so-called social interfaces occur at points where varied and conflicting social fields or life-worlds intersect, forming the stage where power dynamics become manifest. Interface analysis aims to explicate the types and sources of social discontinuity and to explore the cultural and organizational means of transforming or reproducing them [19]. We used this conceptualization because it allows us to identify and characterize the spaces where power relations among actors were exercised [19–21].

Further, to examine the nature of power dynamics that occurred at these interfaces, we employed VeneKlasen and Miller’s [22] *expressions of power* framework. This framework postulates that power is expressed in four main forms namely: 1) power over; 2) power to; 3) power with, and; 4) power within (Table 2) [20, 22]. An advantage of this framework is that it recognizes positive attributes of power and allows actors to view power as something positive that they can possess [5].

**Table 2:** Four expressions and sources of power

Forms of power	Definition
Power over	Power over is exercised by taking it (power) from someone else and using it to dominate and prevent others from gaining it
Power with	Power with is exercised by finding common ground among different interests and building collective strength
Power to act	Power to act refers to the capacity for individuals and groups to shape their life and world and create more equitable relations and structures of power
Power within	Power within refers to individuals’ sense of self-worth, values and self-knowledge which is central to individual and group understanding of being citizens with rights and responsibilities’

### Data analysis

Transcribed data were imported into NVIVO 10 for coding and analysis. Data analysis was led by the first author (EB), with support from all authors. Data were analyzed using a modified framework (thematic) approach. This approach was adopted so as to provide findings and interpretations that are relevant to policy and also to provide pragmatic recommendations. However the approach was modified to include an initial open coding step to allow for emergence of important themes which might not have been captured in the study’s theoretical frameworks (Additional file 2). Even though the coding was carried out by EB, peer debriefing with all the other authors/researchers was carried out to help reduce the possibility of bias. Coded and charted data were critically examined under each thematic category. Interpretation of the data entailed identifying key concepts and explaining relationships between these key concepts. Also, it entailed explaining relationships between the data and theoretical assumptions and identifying messages that are relevant to policy makers. Rigor and trustworthiness were enhanced by a combination of 1) use of theory, 2) use of multiple rather than single case study, 3) prolonged engagement (7 months) during data collection, 4) methodological triangulation, and 5) member checking, which entailed discussing the preliminary findings and interpretations with study participants to verify the validity of our interpretations.

## Results

Far from being a harmonious milieu, the interactions of actors in the case study hospitals resulted in socially constructed interfaces between: 1) senior managers and middle level managers 2) non-clinical managers and clinicians, and 3) hospital managers and the community. Within each interface, we explore actor interactions, highlighting micro-practices of power, the power they possess, how this power is exercised and how this influences PSRA processes.

### Senior managers and middle level managers

The case study hospitals had two management committees namely the hospital management team (HMT) and the executive expenditure committee (EEC). The HMT included middle level and senior hospital managers and reported to the EEC, which included senior level managers only. These managers (senior and middle level) were professionals who had been assigned management duties. The professionals who take on the responsibility of managing professional work, professional colleagues and other staff have sometimes been referred to as hybrid managers [23]. The hospitals did not have clear guidelines on either the roles or the exact composition of the two decision making committees, and neither had an official organogram. In the absence of clarity on roles and composition, these structures and their functioning evolved quite differently in the two case study hospitals. For example, while the Hospital accountant and the hospital pharmacist were considered senior managers and therefore sat in the EEC in Hospital A, they were not considered senior managers and hence only sat in the HMT in Hospital B. Further, while the nursing department was represented in the HMT by the head of the nursing department in Hospital B, the nursing department was represented in Hospital A’s HMT by the head of department and all the nursing-in-charges of each of the wards in the hospital. Also, while the EEC was considered to be the ultimate decision making body with regard to the Hospital Budget in Hospital A, in Hospital B the equivalent was the HMT. Thus, hospital managers are seen to exercise discretionary power to develop hospital guidelines on the roles and composition of decision making committees, with differing results.

In both case study hospitals, the authority vested in the position of senior managers meant that they exercised *power over* the middle level managers. The interactions between these groups of managers resulted in a social interface where power dynamics were manifested, as observed particularly in the budgeting process. However, how this power was exercised differed between the two case study hospitals, as will be argued below.

### Interactions between Senior and Middle Level Managers in Hospital A

In Hospital A, the dominant group of actors in the budgeting and planning processes was the EEC. This committee made the actual allocation decisions and developed final budgets for the hospital. The interface between senior and middle level managers in this hospital was therefore characterized by a power imbalance in favor of the senior managers, who derived and exercised their power over middle level managers in a number of ways.

One of the sources of power of senior managers was their control over resources. Middle level managers felt that the EEC was the most powerful decision making body given that they made the actual allocation decisions:

*“Those [EEC] are the people who actually allocate resources. Once you have that power to allocate resources then you are the most powerful person and you are actually the one who is planning for the whole hospital.” Middle level manager, Hospital A*

Senior managers also derived their power from their positions of authority. The medical superintendent (who was the hospital chief executive), the hospital matron (who was the head of the nursing department), the Hospital administrative officer (who was the head of the administrative staff and non-clinical departments) and the Hospital accountant therefore had significant power by virtue of their positions as senior managers in the hospital. Senior managers therefore exercised *power over* the middle level managers:

*“People can argue and argue but what the medical superintendent says is final because he is the boss. If he says he will give you fifty thousand shillings that is what you will get.” Middle level manager, Hospital A*

*“I think the matron has more powers than other managers…because of her position…the HAO [Hospital Administrative officer] also has a lot of power because he is the one that calls for and chairs the meetings.” Middle level manager, Hospital A*

In addition to *power over*, one of the senior managers in Hospital A, the hospital matron, further derived her power from mobilizing the nursing staff represented in the HMT to support her proposals. She had incorporated all the nursing ward in-charges in the HMT which resulted in the nursing department having the highest number of representatives in the committee. The hospital matron therefore exercised *power with* the other nursing ward in-charges to exert greater influence over allocation decisions:

*“The matron has more power because she has the numbers and is more eloquent……the nurses can be able to make a lot of noise and their proposal is considered, but you are only a single person.” Middle level manager, Hospital A*

Senior managers in Hospital A are seen to exercise power by directly influencing the decision making processes. In addition to these visible forms of power, senior managers in Hospital A also exercised hidden forms of power through their ability to control the decision making agenda. Specifically, they restricted HMT meetings to merely presenting budgetary requests, and deferred the actual budgetary allocation decisions to the EEC. While the HMT held meetings to present budgetary requirements from different hospital departments, the medical superintendent who chaired the meeting always directed that actual budgeting and allocation decisions would be made in the EEC.

*“We [HMT] don’t make decisions…you saw what happens in those meetings. We talk and talk and in the end the decisions are always deferred to the smaller committee [EEC].” Middle level manager, Hospital A*

The power differences between senior and middle level managers in Hospital A were also manifested in the nature of interactions between these two groups during the HMT meetings. We observed that the senior managers were more vocal, while the middle level managers hardly participated in the discussions. We also observed that the organization of the meetings as well as agenda setting was carried out by senior managers only (the Hospital administrative officer, the Hospital accountant and medical superintendent) with no involvement of middle level managers. From our observations of how meetings were conducted, the senior managers sat at a table in front of the room (high table) facing middle level managers who sat in seats arranged in rows (as in a classroom). During the meetings, there was hardly any deliberation and middle level managers appeared reluctant to engage and participate in discussion. Views of senior managers carried the day and the middle level managers were expected to accept decisions without questioning them.

*“It is as if we [middle level managers] don’t matter, they [senior managers] call the meetings, they talk and talk and we just listen. Even if any of us [middle level managers] says anything, it is not taken into consideration.” Middle level manager, Hospital A*

The power imbalance between senior and middle level managers was exacerbated by the fact that two senior managers not only appeared to wield power but also used it for personal advantage. These two senior managers exercised significant influence over hospital decision making. For example, we observed that they had side (informal) meetings before and after each formal decision making meeting, where they agreed on their preferred outcome of the formal meeting. In practice, decisions were made in these side meetings rather than the formal meetings. These two managers therefore seemed to exercise *power with* to exert influence over hospital PSRA decisions.

*“These are very sensitive issues I think he [the medical superintendent] should handle…I think he is overpowered by those two you know when they are the two they support each other.” Middle level manager, Hospital A*

It was felt by the other hospital managers that these two senior managers derived power from having access to crucial information needed for planning and budgeting.

*“They (the two senior managers) are important given that they have information which is useful for budgeting and planning…for example they have information about finances…they are very important members such that if we plan a meeting and they do not come, it will be very difficult to do budgeting and planning.” Middle level manager, Hospital A*

Middle level managers felt that these two senior managers favored their own departments and those of managers with whom they enjoyed good relations. The priority setting process was therefore perceived to unfair. The power enjoyed by these two managers was such that middle level managers felt to have a chance of success, funding proposals needed their support.

*“They [the two senior managers] decide who gets what…mostly it is their departments and their friends’ departments that benefit…it is not fair at all.” Middle level manager, Hospital A*

*“Most of the time you bring up an issue or propose an idea and if those two [senior managers] are not on your side, trust me it won’t go through, it is as simple as that, so the two of them basically have to back an idea and the medical superintendent does not do anything about it....I think he is just overwhelmed.” Middle level manager, Hospital A*

At the interface between senior and middle level managers in Hospital A, the power differences resulted in frustration and reduced motivation among middle level managers. We observed, and indeed was reported by respondents, that the frustration manifested itself in reduced attendance and participation in HMT meetings among middle level managers.

*“Why should we [middle level managers] attend the [budgeting] meetings? Just to rubberstamp their decisions? You attended them…did you see that most of us don’t attend? We are frustrated…and even if we attend, we don’t talk much, because our points are not considered.” Middle level manager, Hospital A*

It also created an atmosphere of suspicion and reduced trust between the middle level managers and senior managers. Middle level managers in Hospital A felt that the budgeting process was not transparent and suspected that some of the senior managers took advantage of the process and perpetrated corruption.

*“There is something going on there…you cannot trust some of them [senior managers]. I think they don’t want us to be very involved in the process [budgeting] because they don’t want us to know what they are hiding.” Middle level manager, Hospital A*

### Interactions between senior and middle level managers in Hospital B

The power dynamics between senior and middle level managers in Hospital B were remarkably different from those in Hospital A. Power differences between these two groups of managers appeared to be reduced or “managed”. Hospital managers in this hospital felt that this was as a result of the medical superintendent’s initiative to ensure PSRA in the hospital was an inclusive and consultative process. The medical superintendent in this hospital exercised her *power to* and *power over* to ensure that all managers were included in the decision making process. Unlike Hospital A, the hospital budget was deliberated on and finalized in the HMT at Hospital B. The medical superintendent felt that it was only by developing the budget at the HMT level that all managers - senior and middle level - would understand and accept the outcome of the process.

*“We try to make sure that all managers are involved in the budgeting process. If the budget is developed by the HMT, then every manager gets a chance to contribute. This way they understand how difficult it is to develop the budget and they understand why they cannot always get what they ask for.” Medical superintendent, Hospital B*

We observed that the atmosphere in the budgeting meetings in Hospital B was less tense than in Hospital A and all managers, whether senior or middle level, felt free to speak out. As a result, middle level managers felt included in the decision making process and thought that the process was fair. The suspicion that accompanied the budgeting and resource allocation process in Hospital A was absent in Hospital B.

*“We [hospital managers] are usually involved in the budgeting process and so there is nothing to hide. All the money collected is announced in the HMT and we decide how to spend it. The process is transparent.” Middle level manager, Hospital B*

### Non-clinical managers and clinicians

Another interface where power differences played out in PSRA practices was between hospital managers in general (hospital administration) and clinicians. In both case study hospitals, clinicians did not participate in budgeting and planning activities for the hospital. The EEC and HMT in both hospitals comprised of non-clinical managers except for the medical superintendent. Even though clinical departments such as obstetrics and gynecology and pediatrics were headed by clinician managers, these departments were represented in these meetings by the nurses rather than the clinician managers. Senior managers felt that one of the reasons clinicians did not participate in budgeting and planning meetings was that they were not interested in participating in management or administrative activities given their focus on their clinical responsibilities.

*“The doctors are just not interested in hospital management issues. They know when we plan for the meetings but they don’t show up because they prefer to be in the wards and the clinics.” Senior manager, Hospital B*

On closer examination, it appeared that one of the major contributors to non-participation of clinicians was professional identity. Clinicians in both hospitals did not seem to think that managerial responsibilities such as budgeting and planning were part of their roles as professionals. They identified themselves more with their clinical roles and considered time spent doing managerial duties as “wasted time”.

*“To be honest, I have never attended the annual work plan meetings….I just feel that that my work is to see patients and not to sit in meetings….so I focus more on that.” Frontline clinician, Hospital A*

*“Most of us [doctors] don’t really see our role in all these management meetings…that is the work of the managers. We don’t see that as working so we are more at home with seeing the patients.” Frontline clinician, Hospital B*

Both senior managers and clinicians felt that the shortage of clinical staff also contributed to the non-participation of clinicians in budgeting and planning meetings.

*“It is just because of time. I am the only anesthetist in this hospital. And there are always, you know, emergencies popping up here and there. So you find that there is a management meeting planned but I still can’t attend because I am attending to an emergency”, Frontline clinician, Hospital B*

This reason was also perhaps not totally honest given that clinicians did not spend all of their time in the hospital but rather dedicated a significant proportion of it to personal/private practice either in their own clinics or neighboring private clinics. They therefore had very little time left to attend to PSRA activities in the hospitals.

*“All these doctors locum in private clinics. The senior ones in fact have their own private clinics in town. So they don‘t want to come for budgeting meetings because it will eat on their time for their private practice.” Middle level manager, Hospital A*

*“They [the doctors] have two jobs, here and in the private hospitals. So they cannot find time to participate in hospital management because they are busy making money in the private hospitals.” Middle level manager, Hospital B*

However, clinicians in both hospitals reported that one of the main reasons they did not participate in PSRA activities was because they were not invited to participate.

*“We [doctors] don’t get invited to most of these meetings….you just come to hear that there was a meeting and budgets were allocated…maybe if I am invited I might find time to attend some of the meetings” Frontline clinician, Hospital A*

It was also evident from their responses that they had poor knowledge of the PSRA activities that take place in the hospital. For example, clinicians interviewed were generally unaware of the decision making structures in the hospital such as the HMT, EEC and HMC. Clinicians felt that they were excluded from PSRA activities and that these activities were controlled by a “clique” of hospital managers:

*“I have never attended an annual operational planning meeting, either because of, you know, sometimes you are not even aware there is a meeting going on, because it seems like there is a certain clique of people who are always involved in those meetings.” Frontline clinician, Hospital A*

Over time, because of this exclusion, clinicians in both hospitals had come to accept their non-participation as the norm. Hospital Administrators hence appeared to exercise invisible power over PSRA processes such that clinicians had come to believe that it was not part of their role or their right to participate in these processes.

*“To be honest the reason why I have not attended some of these meetings is because I have not seen my fellow colleagues attending the meeting. My senior colleagues…because when you report to a certain institution sometimes you tend to do what people do, you follow the norms.” Frontline clinician, Hospital B*

In both hospitals, clinicians appeared to exert more influence over the medicine selection decisions. This was because these decisions required expert knowledge on medicines that the non-clinical managers did not have. For decisions that required clinical knowledge, clinicians were able to exercise their power derived from technical knowledge.

*“Of course the clinicians have an influence. They are the guys who prescribe medicines. Sometimes different clinicians have different preferences for medicines to use for certain conditions. Take me for instance. If I am treating a certain condition, out of experience I know this drug works much better than the other drugs so I’ll request for it to be stocked by the pharmacy.” Senior manager, Hospital A*

*“The doctors determine what medicines the pharmacist will buy. This is because they deal with the patients and so they know what medicines the patients need. So they tell the pharmacist to stock the medicines that they think are needed by their patients.” Frontline clinicians, Hospital B*

At the interface between non-clinical hospital managers and clinicians, the key outcome is the exclusion of clinicians from hospital PSRA processes, with the exception of the medicines selection process. In both case hospitals, this exclusion was thought to result in the misrepresentation of clinical priorities. Further, clinicians thought they had more legitimacy in making priority setting decisions by virtue of their expertise and clinical roles, compared to non-clinician managers.

*“Some of the things are dealt with by the office of the administrator…since he is not a clinician he does not understand what we go through.” Frontline clinician, Hospital B*

Perhaps more importantly, the non-participation of clinicians in PSRA activities meant that only the values of one set of actors (hospital managers; non-clinicians) were influential in decision making. Partly because of the socialization from professional (non-clinical) background and also because of their knowledge (or lack thereof), non- clinical managers placed more value on considerations that had administrative importance. For example, in deciding whether or not to fund a proposal, they were more concerned about whether the hospital could afford to fund the activity, and whether the activity had the potential to generate revenues that could add to the hospital resource envelope, than about whether the service would meet the healthcare needs of the community served by the hospital.

“*The hospital generates very little money which means priorities have to change…So first we want to make money, we allocate where we can make money.” Senior manager, Hospital B*

The managers thus operated under a *fiscal-managerial* decision system [24], as opposed to clinicians’ greater concern about the needs of the patients (regardless of whether they were paying patients or not) and their ability to deliver health care services to these needy patients. Clinicians were also generally more concerned about whether services were effective and of good quality, suggesting they operate under a *medical-individualistic* decision system [24].

*“All they [non-clinical managers] think about is money, money, money…they don’t look at the patient perspective…what the patient needs…quality of care…I think these should be a priority. But they [non-clinical managers] think more of how to raise revenues.” Frontline clinicians, Hospital B*

With clinicians excluded from decision making processes, the *fiscal–managerial* value system dominated PSRA decisions. The dominance of non-clinical manager values resulted in the prominence of the revenue generating potential as a criterion for PSRA in both case study hospitals. Departments or service areas that generated more revenues from user fees received more resources while those that were poor at revenue generation received little or no resources. This resulted in arguably inequitable allocation of hospital resources [25].

*“Since I am allocated a small budget I only procure medicines that I can sell, I cannot buy medicines for children under 5 years because they don’t pay for services.” Middle level manager, Hospital A*

### Hospital managers and the community

Interactions between hospital managers and the community also provided a platform for the exercise of power. In both case study hospitals, PSRA activities were undertaken by hospital staff either as individuals (in the case of nursing allocation and medicine selection) or as committees (the HMT and the EEC) without any community representation. Hospital managers derived their power relative to community members from a number of sources. First, hospital managers had positional authority (they had been appointed by the government to positions with clear influence over hospital management) while the community had no clear role or authority in hospital management issues. Second, hospital managers had control over hospital resources. Third, hospital managers had better access to hospital information than community members.

Hospital managers in both hospitals felt that it would be difficult to include community members in hospital PSRA meetings because they did not possess the technical capacity to contribute effectively.

*“The problem with community members here is that most of them are uneducated. It is difficult for them to take part in decision making because they don’t have much knowledge.” Senior manager, Hospital A*

*“The community members are not well trained to take part in budgeting. It can be very difficult to involve them in such a process.” Middle level manager, Hospital B*

It was also thought that it would be problematic to include community members in PSRA decisions because they would lack objectivity to make allocation decisions and would be biased by their experiences and their healthcare needs.

*“The problem with community members is that they only think of what affects them. If you involve them in decision making, they might just promote things that affect them only.” Senior manager, Hospital A*

In both case study hospitals, the community was, on paper, only represented in the hospital management committee (HMC), which was an oversight committee of the hospital. In both hospitals however there were concerns about the representation of the HMC. It was felt that the members of these committees were not selected through fair and transparent processes but rather based on perceived relationships with local politicians or hospital senior managers.

*“How are the members selected? It depends on who they know. If you are a friend of the politicians or the senior managers in the hospital then you are made a HMC member.” Senior manager, Hospital A*

*“HMC members are cronies of local politicians. That is how people are selected to be in HMC. It is not a transparent process.” Senior manager, Hospital B*

These HMC members were therefore not thought to represent community members. Further, the fact that their selection was influenced by the hospital managers created the potential for indebtedness. This was because HMC members benefited by drawing (financial) sitting allowances and prestige from the position. The HMC’s oversight role also provided them with an opportunity to access information that would enable the committee members to influence and/or benefit from hospital procurement processes. It is thought that the HMC members were careful not to go against the wishes of the hospital managers so as to retain their support for membership in the HMC.

*“They are toothless…they cannot dare challenge EEC decisions. You see, they get meeting allowances and they also get favors like tenders to supply the hospital…so they don’t want to risk that.” Middle level manager, Hospital A*

Therefore, whereas on paper the community members, through the HMC, had an oversight responsibility over the hospital, in practice the HMC was not empowered. In both hospitals, the HMC was seen as passive, merely “rubberstamping” hospital decisions. For example, whereas the HMC was required to review and amend (if necessary) the budget developed by hospitals, in practice they simply approved the budgets without serious scrutiny.

*“The HMC is toothless, they just rubberstamp decisions. They don’t want to step on the toes of the hospital managers because they are the ones that influenced their selection.” Middle level manager, Hospital A*

*“The committee [HMC] cannot ask any questions. They just accept the decisions of the hospital because they are powerless.” Middle level manager, Hospital B*

At the interface between the hospital and the community therefore, the exclusion of the community from PSRA processes meant that community voice was not incorporated in hospital decision making and put to question both the responsiveness of the hospital PSRA practices to community needs and the legitimacy of these processes.

## Discussion

The interactions between actors in the case study hospitals and the micro-practices of power are seen to have significant influence on PSRA practices. Power differences are seen to fracture the social fabric of hospital actors along three social interfaces. We now discuss the power dynamics within each of these interfaces, in relation to literature, and draw implications and recommendations for the improvement of PSRA practices at the meso level.

Senior managers are seen to exercise both visible and hidden forms of power that excludes middle level managers from decision making processes in one of the case study hospitals. The important role that middle level managers play in the implementation of organizational policies and strategies has been recognized in literature [26]. They have been described as the “coordinator between daily activities of the units and the strategic activities of the hierarchy” [27]. In the case study hospitals, these middle level managers are responsible for the implementation of hospital plans and the day to day operations of their respective departments. Power relations between these middle level managers and senior managers in Hospital A were however seen to influence their performance by causing frustration and reducing their motivation to work. This underlies the importance of understanding power dynamics between these two levels of management and seeking to manage it. The power differences between hospital managers are typical of other settings where decision making environments are politically complex and highly hierarchical (Gibson et al. [12]). For example, in Argentina, senior managers with control over the hospital budget had more power than middle level managers (Gordon et al. 2009). The fact that the power dynamics in the second case study hospital were well managed underlies the critical role that leadership should play in balancing interests and managing the power differences between different actors (Reeleder et al. [28]). By proactively promoting a deliberative and inclusive PSRA process, the medical superintendent in hospital B reduced the power differences between senior and middle level managers and ensured that both groups felt included. The outcome of her leadership was a PSRA process that had increased participation, and managers that had trust in the transparency and fairness of the process.

The power differences between clinical and non-clinical managers (hospital administrators) resulted in the exclusion of clinical managers from PSRA processes in both case study hospitals. Of the theories used to explain this tense relationship between clinicians and hospital managers, the theory of managerial dominance over professionals in organizations perhaps resonates best with the observations in the case study hospitals [29]. The theory sees professionals as joining the working class, losing control of their work and becoming the subject of the exploitative discipline of managers who serve the ultimate interests of the capitalist class [29]. Within healthcare, proponents of this theory have argued that evidence of this trend includes the observation that, firstly more and more clinicians are salaried, rather than self-employed, thus losing their economic independence and assuming the wage-labor status of the working class [29]. This is true in the public healthcare sector in Kenya where all clinicians are salaried. Secondly, more and more clinicians practice in bureaucratic organizations with their work becoming subject to the control of management [29]. This is also true in the public healthcare sector in Kenya which is highly hierarchical and bureaucratic. Drawing from concepts of power, managers are able to enforce their dominance through power derived from position (authority), control over resources, coercion, and control over information, amongst other influences [7]. Tensions between clinicians and hospital managers is a recurrent theme in studies of healthcare organizations [30–32]. Specifically, it has been reported in other settings that physicians were marginally involved in PSRA practices. For example, in a Ugandan hospital, it was reported that the PSRA process had minimal involvement of frontline staff and was dominated by hospital managers [33, 34]. Power struggles between frontline practitioners and managers who were reluctant to share decision-making power, and frustration by practitioners when their concerns were not addressed, were reported to contribute to the non-participation of practitioners [33]. It has been demonstrated that the involvement of clinicians in decision making processes improves healthcare decision making [35, 36]. While managers and clinicians have differing values and interests, both are important, and finding a balance between them is crucial in improving decision making in healthcare [30].

Power relationships are also seen at the interface between the hospital managers and the community, with the latter minimally involved in PSRA processes. In this case professionals exercise power derived from the sources described above to minimize the involvement of community members. The minimal involvement of the community and patients in the case study hospitals was attributed in part to community members and patients being perceived to lack understanding and objectivity. Minimal involvement of the community in PSRA processes for these reasons has also been observed in other settings [37]. There is increasing recognition however for the need to incorporate the public in PSRA decision making [38], with community involvement considered to strengthen legitimacy of the process and system accountability. The views and the needs of the community are also arguably better represented if the public is involved. The incorporation of community views is however not without challenges. These include difficulties in defining communities, identifying legitimate representatives, lack of role clarity, low capacity to participate effectively, and few resources to facilitate engagement mechanisms, among others [39, 40].

These findings reinforce the political frame which views organizations as coalitions comprised of individuals and groups with enduring differences in values and interests, living in a world of scarce resources, where power and conflict are at the center of organizational decision making [7]. It emphasizes the importance of getting the institutional settings right, so as to ensure that PSRA processes are not dominated by any particular interest and that the relevant set of actors effectively contribute to decision making. In the case of public hospitals in Kenya, a first step would be to develop clear decision making structures and enhance clarity about the roles of these structures. The lack of role clarity meant that while actual budgeting decisions were made in the EEC in one of the case study hospitals, they were made in the HMT in the second case study hospital. Given that the EEC was comprised of a small group of senior managers, while the HMT was comprised of a larger group of both senior and middle level managers, the latter is considered a more inclusive forum for decision making. It has been shown however, that inviting the relevant range of actors into decision making processes does not necessarily translate into effective involvement [41]. There is still the need to put in place measures that ensure that these actors are empowered to participate. First, there should be explicit requirements that allocation decisions can only be made if a specified quorum of relevant actors (senior managers, middle level managers, frontline clinicians and community representatives) is represented. Second, the institutional setting should be such that each stakeholder has equal opportunities (through representation) to participate at different stages of the decision making process; these include establishing procedural rules, agenda setting, selecting the expertise and information to inform the process and providing an assessment of the validity of information. Third, the roles of each stakeholder should be clearly defined and enshrined in PSRA rules and guidelines. Fourth, relevant information should be accessible to each stakeholder to reduce information asymmetries. Finally, there should also be ongoing rather than one off or infrequent engagement of stakeholders since it has been shown that ongoing engagement builds trust over time [42, 43]. To make these recommendations more practical, participation by stakeholders could be through representation. For example, it would be more practical to have a representative of different cadres of hospital staff, such as doctors, rather than all doctors in the hospital participate in budgeting processes. Further, the extent of stakeholder engagement and involvement could be adapted to the nature and scope of PSRA decisions. For example, major PSRA processes such as hospital strategic planning or budgeting would require wider stakeholder engagement compared to staff allocation decisions within a hospital department.

Beyond developing structures that minimize power differences and encourage inclusive processes, these findings also highlight the importance of agency. Power differences were minimized in Hospital B largely because the hospital superintendent encouraged inclusiveness and deliberation among actors in the hospital PSRA process. Strengthening the leadership in hospitals is therefore also an important intervention [25]. An important role of leadership in healthcare settings is managing actor relations and balancing power dynamics in decision making processes so as to ensure that every actor has a chance to participate [28]. Skills such as the ability to mentor and motivate staff, awareness and appreciation of deliberative processes, the ability to manage relationships and build trust among actors should therefore be surfaced as important skills for hospital managers. It is unlikely that these “soft skills” will be developed by the formal “classroom type” leadership and management trainings [44]. These skills can be better learnt through doing, reviewing the situation carefully and working on how the learning can be built on for next time [45]. One approach that has been proposed as a tool for the development of soft leadership skills is action or collaborative learning [44, 46]. Action learning programmes are typically tailored to focus on the issues facing the organization and combine formal training with on the job mentoring, employing assignments and reflections from work experiences to achieve learning [47]. In this approach, learning is not transmitted from teacher to student but rather co-produced by the interactions and engagements between the facilitators and the leaders with real life situation. There are still questions however about the scalability and affordability of such initiatives [44]. Another approach that has been promoted as a tool for leadership development is coaching. Leadership coaching has been defined as “a helping relationship formed between a client who has managerial authority and responsibility in an organization and a coach who uses a wide variety of behavioral techniques and methods to help the client achieve a mutually identified set of goals to improve his or her professional performance and personal satisfaction, and consequently to improve the effectiveness of the client’s organization within a formally defined coaching agreement” [48]. It is an interactive process that provides a safe place for reflection and learning, thereby enabling empowerment, learning and performance improvements [49].

## Conclusion

In this paper we have analyzed and presented the influence of micro-practices of power in PSRA processes using two public hospitals in Kenya as case studies. The findings emphasize the need to put in place institutional measures to minimize the effect of power differences among the relevant range of actors in PSRA processes. They also emphasize the need to develop the leadership skills of hospital managers, beyond hard skills such as planning, budgeting, and organization, to include soft skills that enable them to motivate and build trust among staff, promote deliberative processes and manage power dynamics among actors in hospital PSRA processes. Further, the mechanisms of engaging the community in hospital priority setting decisions should be strengthened, as well as the communities capacity to effectively participate in decision making.

## Additional files

Additional file 1: Questions used to explore power. (DOCX 93 kb) Additional file 2: Sample coding tree and coding process. (DOC 40 kb)

## Abbreviations

AWP: Annual work plan
EEC: Executive expenditure committee
GOK: Government of Kenya
HAO: Hospital administrative officer
HMC: Hospital management committee
HMT: Hospital management team
MOH: Ministry of health
MTC: Medicines and therapeutic committee
PRSA: Priority setting and resource allocation
