# Supplementary material for: The influence of power and actor relations on priority setting and resource allocation practices at the hospital level in Kenya: a case study
Source: BMC Health Serv Res. 2016 Sep 30;16:536. doi: 10.1186/s12913-016-1796-5 (PMC5045638; doi:10.1186/s12913-016-1796-5)
Supplement: Additional file 1: — Questions used to explore power. (DOCX 93 kb) [file 12913_2016_1796_MOESM1_ESM.docx]

**Additional File 1: Data collection questions used to explore power dynamics in priority setting processes**

| **Data collection method** | **Guiding questions** |
| --- | --- |
| **In-depth interview topic guide questions** | - In your opinion, who influences the budgeting process in the hospital? How do they influence this? - In your opinion, who influences in the annual work planning process in the hospital? How do they influence this? - In your opinion who should be involved in the budgeting process in the hospital? why? Who should not be involved? why? - In your opinion who should be involved in the annual work planning process in the hospital? Why? Who should not be involved? why? - Who is actually involved? why? - What are the roles of each of the actors? - What are the interests of each of the actors? - At what stage of the budgeting/annual work planning process is each of the actors involved? - What is the role of each or the actors/participants in the decision making process? - What is the relationship between the actors you have mentioned? |
| **Non-participant observations checklist** | - Which people or groups of people take part in the priority setting process? - Which actors participate in decision making? - How do actors relate during meetings and also out of meetings? - Whose voice is heard most in meetings, whose isn‘t ? - Who seems to influence decisions more than others? - What is the relative influence of different actors in the decision making process? - Who has the final decision making power? |
| **Document review checklist** | - Who calls for meetings? - Who attends meetings? - Who contributes during meetings? |
