# Supplementary material for: The influence of power and actor relations on priority setting and resource allocation practices at the hospital level in Kenya: a case study
Source: BMC Health Serv Res. 2016 Sep 30;16:536. doi: 10.1186/s12913-016-1796-5 (PMC5045638; doi:10.1186/s12913-016-1796-5)
Supplement: Additional file 2: — Sample coding tree and coding process. (DOC 40 kb) [file 12913_2016_1796_MOESM2_ESM.doc]

**Example of the coding process – Actors, their power and interest**

**Direction of the Process**

| **ACTORS, THEIR POWER AND INTEREST** | | |
| --- | --- | --- |
| **Step 3- Axial coding - aggregation of sub-themes into overarching themes**  ***(axial codes informed by literature, conceptual framework, sub themes)*** | **Step 2 – refinement of open codes to sub themes**  ***(Sub-themes informed by open codes and literature)*** | **Step 1 – Open Coding Process -lines, sentences, paragraph coding** |
| **Range of Actors** | Who are the actors | The two managers seem to have more power than the med sup  The disproportionate power that the HOA and accountants has led to demotivation of other staff  The disproportionate power that the HAO and accountant have resulted in a lack of trust  Sources of power  The HAO and accountants power are in part as a result of the med sup being too busy  Provincial office rubberstamps decisions  Priorities from clinical departments overlooked because clinicians don’t participate in planning  Power used to favor departments  Power plays due to position and professional identity  Power differences demotivates other managers from participating in planning meetings  Only two individuals make decisions  Mistrust is impeding the working together among decision makers  Med sup favors his department-surgery  Managers think planning meetings are a waste of time  Managers skip planning and budgeting meetings because they don’t get allocations and are hence frustrated  Managers skip planning and budgeting meetings because their input is overlooked  Managers don’t participate in planning meetings because they are used as avenues to advance personal interests and fights  Managers don’t participate in planning meetings because of negligence  HMT members are not empowered to question what happens in the hospital  HMT members are demoralized because of the decisions of the EEC  HAO and the accountant misuse their powers  HAO and the accountant have disproportionate power when compared to other managers  Feeling that EEC should be disbanded  Feeling that Accountant and HAO are involved in corruption  Feeling by medics that while admin has power they don’t understand clinical needs  EEC members favor their own departments over others  Donors not involved in planning and budgeting meetings  Discontent over the power of the accountant  Coz the accountant has power their priorities take precedence over others  Composition of the HMC finance committee  Clinicians skip planning meetings because they feel represented by the medical superintendent  Clinicians lack understanding and knowledge of the hospital planning processes  Clinicians identify with the hospital medical superintendent because he is a clinician too  Clinicians have not received management training  Clinicians have delegated management roles to the nurses  Clinicians feel they need to be part of the hospital planning process  Clinicians feel they are blamed by patients for lack of essential supplies  Clinicians feel frustrated by lack of implementation of their proposals  Clinicians feel frustrated by lack of essential supplies  Clinicians don’t participate  Clinicians don’t attend planning meetings due to frustration because their input is overlooked  Clinicians don’t attend planning meetings because they are not invited to meetings  Clinicians don’t attend planning meetings because they are few and busy  Clinicians don’t attend planning meetings because of negligence  Clinicians don’t attend planning meetings because it is part of hospital culture  Clinicians do not participate in hospital planning and budgeting  Clinicians bear the responsibility of explaining to patients the lack of essential supplies  Actors and their power  Account and HAO make decisions without communication to the rest of the team  HMT members are disempowered |
|  | What is their role |  |
|  | Actor participation in planning and budgeting activities |  |
|  | How do the actors exercise their role |  |
| **Actor Power** | Levels of power |  |
|  | Spaces of power |  |
|  | Sources and forms of power |  |
|  | Exercise of power |  |
|  | Effect of power to priority setting |  |
|  | Effect of power to actor relations |  |
|  | Effect of power relations to planning and budgeting activities |  |
|  |  |  |
| **Actor Interest** | Actor values |  |
|  | What is important to each actor |  |
|  | How do actor values interact with priority setting |  |
